# Risk factors for recurrent severe anemia among previously transfused children in Uganda: an age-matched case-control study

**DOI:** 10.1186/s12887-019-1398-6

**Published:** 2019-01-18

**Authors:** Aggrey Dhabangi, Richard Idro, Chandy C. John, Walter H. Dzik, Robert Opoka, Ronald Ssenyonga, Michael Boele van Hensbroek

**Affiliations:** 1 Child Health and Development Centre, Makerere University College of Health Sciences, Mulago upper hill road, P O Box, 6717 Kampala, Uganda; 2 Department of Pediatrics and Child Health, Makerere University College of Health Sciences, Kampala, Uganda; 3 Ryan White Centre for Pediatric Infectious Disease and Global Health, Indiana University School of Medicine, Indianapolis, IN USA; 4 Department of Pathology (Transfusion), Harvard University / Massachusetts General Hospital, Boston, MA USA; 5 Clinical trials unit, Department of Epidemiology and Biostatistics, School of Public Health, Makerere University College of Health Sciences, Kampala, Uganda; 6 Department of Global Child Health, Emma Children’s Hospital, Academic Medical Centre, University of Amsterdam, Amsterdam, The Netherlands

**Keywords:** Recurrent severe anemia, Children, Transfusion, Malaria, Hemoglobinuria, Sickle cell anemia

## Abstract

**Background:**

In resource-poor settings, transfused children often experience recurrence of severe anemia (SA) following discharge from hospital. This study determined the factors associated with recurrent severe anemia (RSA) among previously transfused Ugandan children aged less than 5 years.

**Methods:**

A case-control study was conducted in five hospitals in Uganda from March 2017 to September 2018. We prospectively enrolled 196 hospitalised children who had been transfused for severe anemia 2 weeks to 6 months prior to enrollment. Of these, 101 children (cases) were re-admitted with a hemoglobin [Hb] level of ≤6 g/dL and required transfusion; and 95 children (age-matched controls) were admitted for other clinical illness with a Hb > 6 g/dL. Children known to have sickle cell anemia, cancer, or bleeding disorders were excluded. Clinical and laboratory evaluation were done. Conditional logistic regression adjusted for age, was used to determine factors associated with RSA.

**Results:**

The median time (IQR) between the earlier transfusion and enrollment was 3.5 (1.9–5.7) months for cases, and was 5.0 (2.9–6.0) months for controls (*p*-value = 0.015). Risk factors (adjusted odds ratio, 95% confidence interval, and significance) for development of RSA were: hemoglobinuria (36.33, 2.19–600.66, *p* = 0.012); sickle cell anemia – newly diagnosed (20.26, 2.33–176.37, *p* = 0.006); history of earlier previous transfusions (6.95, 1.36–35.61, *p* = 0.020) and malaria infection (6.47, 1.17–35.70, *p* = 0.032).

**Conclusion:**

Malaria chemoprevention, follow up visit for Hb check after discharge from hospital and sickle cell screening among previously transfused children represent practical strategies to prevent and identify children at risk for recurrent severe anemia. The cause of hemoglobinuria in children merits further investigations.

## Background

Recent studies have indicated that up to 10% of children who receive blood transfusion for severe anemia (SA) return to health facilities with recurrence of severe anemia within three months of discharge, while others die at home [1]. Moreover, children with recurrent severe anemia (RSA) are 10 times more likely to die compared to their non-anemic counterparts during the post-discharge period [1]. However, the risk factors associated with recurrent severe anemia among previously transfused children have not been sufficiently studied.

Malaria infection in the immediate post-discharge period has been identified to be a major contributing factor to RSA in children [2]. Other documented risk factors include; poor socio-economic status, large family size, history of recurrent transfusions and human immuno-deficiency virus (HIV) infection [1]. Recurrent life-threatening anemia in children in sub-Saharan Africa may have several underlying mechanisms. From a pathophysiological view point, the relative roles of insufficient erythroid production and increased red cell destruction have not been fully defined [3, 4].

A better understanding of the risk factors for development of RSA can identify children at highest risk and can enhance the benefits of blood transfusion in children [5, 6]. We conducted an age-matched case-control study to determine the factors associated with recurrent severe anemia among previously transfused Ugandan children aged less than 5 years.

## Methods

### Study design

This was an age-matched study with a ratio of 1:1 between cases and controls.

### Study setting

The study was conducted at Jinja, Masaka, Hoima, Mubende and Kamuli hospitals, in Uganda. The first four are all public regional referral hospitals serving east-central, south-central, north-central, and western sub-regions of Uganda respectively, while Kamuli mission hospital is a private not-for-profit hospital located in eastern Uganda. Each of the hospitals has a pediatric ward with an in-patient bed capacity ranging from 30 to 80.

### Study population, inclusion and exclusion

We enrolled children aged 2 months to 5 years with prior severe anemia (cases and controls) that required a blood transfusion, which was given > 2 weeks but < 6 months prior to enrollment in the present study. Cases were defined as children who at the time of study enrollment were re-admitted to the hospital with a hemoglobin level of ≤6 g/dL and required blood transfusion. Controls were defined as children who at the time of study enrollment were being seen for other clinical illness as an inpatient or outpatient, and had a hemoglobin level of > 6 g/dL. Cases and controls were matched using an age range of ±12 months. Children known to have sickle cell anemia (SCA), cancer, bleeding disorders or whose anemia was caused by trauma, were excluded.

### Sample size and sampling

The sample size of 196 was estimated using Open EPI calculator; using malaria as the main risk factor with a prevalence of 29.3% among cases and 12.1% among controls, according to a study by *Phiri KS* et al 2008 [1], and considering a two-sided confidence level of 95%, an acceptable type 1 error of 5% and power of 80%. A study clinician evaluated all prospective participants for eligibility at each study site and eligible participants were enrolled consecutively.

### Study variables and data collection

Clinical evaluations included the past medical history (including the number of previous transfusions, past hospitalizations and diagnosis during these hospitalizations), socio-family history and a detailed physical examination and anthropometric measurements (weight, height and mid-upper arm circumference [MUAC]). Socio-demographic data collected included; sex, age in months, age and occupation of caregiver, number of household members, number of children in the household, number of meals per day, among others. A structured case-report form was used to record study variables.

### Laboratory measurements

A blood sample of 2 ml collected in an EDTA tube was taken (for cases - taken off at pre-transfusion). Hemoglobin was measured using a point-of-care device (Hemocue® 201, Angelholm, Sweden). ABO typing and Rhesus blood grouping was done using commercial reagents available at the hospital blood banks. Complete blood count (FBC) tests were performed using the Mindray automated haematology analyzer (Shenzhen Mindray Bio-Medical electronics Co. Ltd., Shenzhen, China). Sickle cell status (either Hb-AA, AS or SS) was determined by capillary hemoglobin electrophoresis assay (Sebia minicap, Evry-France). For patients returning within two months since previous transfusion, hemoglobin electrophoresis was deferred for at least two months. HIV serology was tested using HIV-1/2 test strips (Alere Medical Co. Ltd., Chiba, Japan). Urine analysis was done using URS-10 T reagent test strips (Zhejiang Orient Gene Biotech Co. Ltd., Zhejiang, China). Malaria thick-smear was stained using field stain A and B. Malaria rapid diagnostic test (RDT) was done using SD Bio-line malaria Ag P.f/Pan test strips (SD Standard diagnostics, INC, Alere Co., Korea). A reticulocyte count (%) was performed from a fresh (within 2 h) sample, using a thin smear stained with new methylene blue, and the reticulocyte production index – RPI (corrects for the degree of anemia) was calculated using the method of *Poorana* et al [7]. Some tests such as Hb-electrophoresis and reticulocyte count could not be performed among some urgent cases who presented in the night and for whom a pre-transfusion study sample was not obtained.

The presence of asexual forms of plasmodium species on a thick malaria smear or a positive malaria RDT defined the diagnosis of malaria, while hemoglobinuria was defined by both a history of passage of dark or red-colored urine, and confirmed evidence of ‘blood’ at urine dip-stick. Sickle cell anemia and sickle cell trait were defined as the presence of Hb-SS and Hb-AS, respectively. Suspected bacteremia was a clinical diagnosis backed by laboratory evidence neutrophilia on FBC. Mean cell volume (MCV) < 70 fL defined microcytosis, while MUAC of ≤12.5 cm defined malnutrition (severe acute, and moderate acute malnutrition) [8].

### Data management and statistical analysis

Data were entered into EPI-DATA version 3.1 software package (The EpiData Association, Odense, Denmark) and analysed using STATA v14.0 (Stata, College Station, TX, USA). We computed descriptive statistics and present, medians (interquartile range), proportions for the demographic characteristics by case or control status. Association between categorical variables was assessed using odds ratios and statistical significance determined using the McNemar test. Means of symmetrical continuous variables were compared using the paired t-test. The difference in median time from earlier transfusion to enrollment was evaluated with a Wilcoxon rank sum test. 95% test-based confidence intervals for odds ratios and *p*-values are presented from conditional logistic regression adjusted for children’s age as matching variable. The stepwise backward model building technique was followed to identify significant factors after adjusting for factors with *p* values < 0.2 for consideration into the multivariable model. A *p* < 0.05 was considered statistically significance. All *p*-values presented are two sided.

## Results

A total of 101 cases and 95 age-matched controls enrolled in the study were included in the analysis. Kamuli study site had slightly more cases than controls (Table 1). The baseline characteristics of cases and controls were comparible except for the median time (IQR) from prior transfusion to enrollment which was 3.5 (1.9–5.7) and 5.0 (2.9–6.0) months among cases and controls respectively (*p*-value = 0.015) .

**Table 1:** Baseline characteristics of study participants

Variable	Study category		
	Total (*n* = 196)	Cases (*n* = 101)	Controls (*n* = 95)
Age of child in months (matching criterion), mean (SD)*	31.3 (14.4)	32.2 (14.3)	30.3 (14.5)
Sex, *n* (%)			
Female	81 (41.3)	43 (42.6)	38 (40.0)
Male	115 (58.7)	58 (57.4)	57 (60.0)
Mother’s age in years, mean (SD)	28.4 (6.3)	28.5 (6.4)	28.3 (6.2)
Study site, *n* (%)			
Jinja	116 (59.2)	58 (57.4)	58 (61.1)
Masaka	34 (17.4)	16 (15.8)	18 (18.9)
Hoima	25 (12.8)	12 (11.9)	13 (13.7)
Kamuli	15 (7.6)	12 (11.9)	3 (3.2)
Mubende	6 (3.1)	3 (2.9)	3 (3.2)
Caregiver relationship to the child, *n* (%)			
Mother	130 (66.3)	67 (66.3)	63 (66.3)
Father	47 (23.9)	26 (25.7)	21 (22.1)
Others (Grand, Auntie, etc)	19 (9.7)	8 (7.9)	11 (11.6)
Occupation of caregiver, *n* (%)			
Employed	48 (24.5)	25 (24.8)	23 (24.2)
Un-employed	148 (75.5)	76 (75.2)	72 (75.8)

*SD = standard deviation

### Matched bivariable analysis

The factors that were independently associated with RSA are summaried in Table 2. History of earlier previous blood transfusions (in the period > 6 months), history of other previous admissions, passage of dark or red-colored urine and Artemisinin-based combined therapy (ACTs) use prior to admission on the current illness were significantly associated with recurrence of SA. A diagnosis of malarial anemia at the most recent previous admission, diagnosis of malaria at the current admission, SCA, and hemoglobinuria were independently associated with RSA.

**Table 2:** Bivariable associations with recurrent severe anemia

Variable	Study category			
	Cases N = 101	Controls N = 95	*Crude odds ratio (95% CI)	*p*-value
Socio-demographics				
Age in months, mean (SD)	32.2 (14.3)	30.3 (14.5)		
Sex, *n* (%)				
Female	38 (40.0)	43 (42.6)	1.00	
Male	57 (60.0)	58 (57.4)	0.92 (0.45,1.85)	0.805
Occupation of caregiver, *n* (%)				
Un-employed	76 (75.2)	72 (75.8)	1.00	
Employed	25 (24.8)	23 (24.2)	0.94 (0.43,2.04)	0.879
Highest education level of mother, *n* (%)				
None and primary	78 (77.2)	66 (69.5)	1.00	
Secondary and above	23 (22.8)	29 (30.5)	0.88 (0.42,1.84)	0.727
Mother’s age, *n* (%)				
≤ 28 years	51 (56.0)	46 (54.8)	1.00	
≥ 29 years	40 (44.0)	38 (45.2)	0.58 (0.66,1.27)	0.173
No. of children in household, *n* (%)				
≤ 2	24 (23.8)	22 (23.2)	1.00	
≥ 3	77 (76.2)	73 (76.8)	0.90 (0.40,2.01)	0.802
Total no. of people in household, *n* (%)				
≤ 7	61 (60.4)	64 (67.4)	1.00	
≥ 8	40 (39.6)	31 (32.6)	1.05 (0.52,2.11)	0.895
No. of meals per day, *n* (%)				
≤ 2	25 (41.7)	29 (67.4)	1.00	
≥ 3	35 (58.3)	14 (32.6)	1.73 (0.67,4.50)	0.258
History				
Passage of dark or red-colored urine, *n* (%)				
No	61 (60.4)	90 (94.7)	1.00	
Yes	40 (39.4)	5 (5.3)	14.7 (3.34,64.80)	< 0.001
ACTs use prior to admission, *n* (%)				
No	66 (65.5)	79 (83.2)	1.00	
Yes	35 (34.7)	16 (16.8)	2.67 (1.21,5.91)	0.015
Malarial anemia at most recent admission				
No	25 (25.0)	18 (18.9)	1.00	
Yes	75 (75.0)	77 (81.1)	0.39 (0.15,0.98)	0.044
History of other previous admissions				
No	36 (35.6)	59 (62.1)	1.00	
Yes	65 (64.4)	36 (37.9)	2.96 (1.59,5.52)	< 0.001
History of earlier previous transfusions				
No	52 (51.5)	78 (82.1)	1.00	
Yes	49 (48.5)	17 (17.9)	4.32 (2.15,8.82)	< 0.001
At current admission				
Malaria diagnosis				
No	33 (32.7)	45 (47.4)	1.00	
Yes	68 (67.3)	50 (52.6)	2.19 (1.08,4.45)	0.030
Suspected bacteremia diagnosis				
No	55 (54.5)	67 (70.5)	1.00	
Yes	46 (45.5)	28 (29.5)	1.93 (0.97,3.84)	0.060
Hemoglobinuria diagnosis				
No	63 (62.4)	90 (94.6)	1.00	
Yes	38 (37.6)	5 (5.3)	12.18 (3.40,43.65)	< 0.001
ǂ Sickle cell status, newly diagnosed				
Normal (Hb-AA)	47 (73.4)	42 (84)	1.00	
Sickle cell trait (Hb-AS)	8 (12.5)	2 (4)	1.44 (0.24,8.71)	0.692
Sickle cell anemia (Hb-SS)	9 (14.1)	6 (12.0)	4.21 (1.03,17.13)	0.045
Reticulocyte production index				
> 2	20 (37.7)	14 (37.8)	1.00	
≤ 2	33 (62.3)	23 (62.2)	1.40 (0.37,5.22)	0.621
MUAC, n (%)				
≤ 12.5 cm	9 (9.1)	5 (5.3)	1.00	
> 12.6 cm	90 (90.9)	89 (94.7)	0.41 (0.10,1.73)	0.223
ABO blood grouping				
Group O	36 (50.0)	28 (50.0)	1.00	
^†^Non- group O	36 (50.0)	28 (50.0)	1.02 (0.41,2.57)	0.963
MCV^¶^				
> 70 fl	41 (100)	36 (94.7)		
≤ 70 fl	0 (0)	2 (5.3)		

* OR by McNemar method, ǂ114 samples tested, ^†^Group A = 28, B = 30, AB = 6 among 128 tested, no stat test done here

In contrast, socio-economic factors such as occupation of the caregiver, highest education level of the mother, number of children in the household, number of meals per day among others, were not associated with RSA.

Other diagnoses such as severe pneumonia, diarrhea, and urinary tract infections were infrequent. Only one participant (a control) was HIV infected.

### Multivariable analysis

All variables with a *p*-value < 0.2 at bivariable analysis (hemoglobinuria, sickle cell anemia, history of earlier previous blood transfusions [in the period > 6 months], passage of dark or red-colored urine, malaria diagnosis at current admission, mother’s age, ACTs use prior to admission, malarial anemia at most recent admission, history of other previous admissions, and suspected bacteremia) were entered into a stepwise backward conditional logistic regression model. After controlling for all ten variables, we found that hemoglobinuria, sickle cell anemia (Hb-SS), history of earlier previous blood transfusions (in the period > 6 months), and malaria diagnosis were each significantly associated with RSA (Table 3).

**Table 3:** Multivariable results for factors associated with recurrent severe anemia

Variable	Crude odds ratio (95% CI)	*p*-value	Adjusted odds ratio (95% CI)	*p*-value
History of earlier previous transfusion				
No	1.00			
Yes	4.32 (2.15,8.82)	< 0.001	6.95 (1.36,35.61)	0.020
Hemoglobinuria at admission				
No	1.00			
Yes	12.18 (3.40,43.65)	< 0.001	36.33 (2.19,600.66)	0.012
Sickle cell status				
Normal (Hb-AA)	1.00			
Sickle cell trait (Hb-AS)	1.44 (0.24,8.71)	0.692	16.10 (0.06,4766.8)	0.325
Sickle cell anemia (Hb-SS)	4.21 (1.03,17.13)	0.045	20.26 (2.33,176.37)	0.006
Malaria diagnosis at admission				
No	1.00			
Yes	2.19 (1.08,4.45)	0.030	6.47 (1.17,35.70)	0.032

### Relationship between hemoglobinuria and sex

There were 10 (23.3%) cases and 1 (2.6%) control female participants compared to 28 (48.3%) cases and 4 (6.9%) control male participants with hemoglobinuria respectively. However at logistic regression, the interaction between hemoglobinuria and sex was not statistically significant. The odds of a participant with hemoglobinuria being male were; AOR = 1.27(95% CI: 0.09–16.88, *p*-value =0.855) that of females.

## Discussion

This study set out to determine the factors associated with recurrent severe anemia among previously transfused Ugandan children aged < 5 years re-admitted to hospital. The results of this study suggest that hemoglobinuria, sickle cell anemia, a history of earlier previous transfusions (in the period > 6 months) and malaria are risk factors for recurrent severe anemia. In addition, history of other previous admissions, and Artemisinin-based combined therapy (ACTs) use prior to admission on the current illness are independently associated with RSA. In contrast, mother’s age ≥ 29 years and a diagnosis of malarial anemia at the most recent admission seem protective. These findings are comparible to the findings of *Phiri KS* et al 2008 and *Lackritz EM* et al 1997 with regard to malaria and history of earlier previous transfusions [1, 2].

It is worth noting that 75.0% of cases had a diagnosis of malarial anemia at the most recent prior admission – in the immediate past six months, before returning with RSA. This underscores the role of malaria in the etiology of severe anemia among children in malaria endemic areas [9]. Although children known or suspected to have SCA were excluded at enrolment, we found 15 children with sickle cell anemia. These had not been diagnosed before. Indeed, in such settings as this with a documented prevalence of sickle cell gene as high as 17% [10, 11], children presenting with SA deserve to be evaluated for SCA.

This study has found hemoglobinuria (defined by history of passage of dark or red-colored urine and the presence of blood at urine dip-stick) to be associated with RSA. This syndrome whose cause is not well understood has recently been documented to be common in the eastern region of Uganda. The syndrome has been associated with SA and positive malaria RDT (despite having negative malaria smears) but not with sickle cell disease or G6PD-deficiency [12]. Although G6PD-deficiency, a sex-linked disorder has been documented to be associated with hemoglobinuria, the study by *Olopot-Olupot P* et al did not find this to be so.

Similarly, in our case-control although we did not perform G6PD assays, we found that the odds of a participant with hemoglobinuria being male were not statistically significant.

The potential relationship between hemoglobinuria and prior use of ACTs and/or other genetic factors in the causation of RSA need to be confirmed. Other reports have shown the possible role of ACTs-related delayed hemolytic anemia in causing RSA [13].

Although the reticulocyte production index was not statistically lower among cases, the potential role of insufficient erythrocyte production predisposing to RSA cannot be excluded for reasons of the fewer numbers we tested. What also remains unknown is how malarial anemia at the most recent admission may be protective against RSA, yet malaria itself is implicated in causing both SA and RSA. This paradoxical finding has also been reported by *Phiri KS* et al 2008.

Nutritional status as measured by the MUAC and socio-economic factors such as occupation of caregiver, highest education level of mother, number of children in the household, number of meals per day, among others were not associated with recurrent severe anemia. Although this study did not have power to evaluate them, there is evidence to suggest that these factors among others may play a significant role in causing both SA and RSA [1, 14]. Contrary to the findings of *Phiri KS* et al where HIV infection was associated with RSA, we found only one participant – a control to be HIV positive. This may be explained by the marked progress made with regard to elimination of mother to child transmission of HIV in Uganda [15].

In summary, recurrent severe anemia among previously transfused children in Uganda occurs after about three months, and is related to hemoglobinuria, sickle cell anemia, history of earlier previous transfusions and malaria infections and/or re-infection.

### Limitation

The current list of factors associated with RSA may not be complete. One uncertainty may be the role of socio-demographic factors such as occupation of caregiver, number of household members, number of meals among others that our study did not have power to evaluate. Similarly, the fewer numbers tested for variables such as reticulocyte production index further limits the power.

### Conclusions

Evidence based interventions are needed to prevent and mitigate the problem of recurrent severe anemia among children. The post-discharge malaria chemoprevention trial (*NCT02671175*) is currently testing the hypothesis that malaria is a key factor in the cause of post-discharge mortality and morbidity in children with severe anemia. The results of that trial are eagerly awaited. However, there is need to address the other risk factors for RSA; such as screening for SCA among all children with SA and a follow up visit between 2 weeks and 3 months for Hb check after discharge from hospital (the best timing for this check remains yet to be determined). Generally, the problem of recurrent severe anemia among children merits further investigation, including areas such as the cause of hemoglobinuria and its potential relationship with prior ACTs use.

## Abbreviations

ACTs: Artemisinin-based combined therapy
EDTA: Ethylene diamine tetra-acetic acid
FBC: Complete blood count
G6PD- deficiency: Glocose-6-Phosphate dehydrogenate deficiency
HIV: Human immuno-deficiency virus
MCV: Mean cell volume
MUAC: Mid-Upper Arm Circumference
RDT: Rapid diagnostic test
RSA: Recurrent severe anemia
SA: Severe anemia
SCA: Sickle cell anemia
